# Overexpression of Glycolate Oxidase Confers Improved Photosynthesis under High Light and High Temperature in Rice

**DOI:** 10.3389/fpls.2016.01165

**Published:** 2016-08-04

**Authors:** Li-Li Cui, Yu-sheng Lu, Yong Li, Chengwei Yang, Xin-Xiang Peng

**Affiliations:** ^1^State Key Laboratory for Conservation and Utilization of Subtropical Agro-Bioresources, College of Life Sciences, South China Agricultural UniversityGuangzhou, China; ^2^College of Life Sciences, South China Normal UniversityGuangzhou, China

**Keywords:** glycolate oxidase, photosynthesis, hydrogen peroxide (H_2_O_2_), salicylic acid (SA), rice (*Oryza sativa* L.)

## Abstract

While glycolate oxidase (GLO) is well known as a key enzyme for the photorespiratory metabolism in plants, its physiological function and mechanism remains to be further clarified. Our previous studies have shown that suppression of GLO in rice leads to stunted growth and inhibited photosynthesis (Pn) which is positively and linearly correlated with decreased GLO activities. It is, therefore, of interest to further understand whether Pn can be improved when GLO is up-regulated? In this study, four independent overexpression rice lines, with gradient increases in GLO activity, were generated and functionally analyzed. Phenotypic observations showed that the growth could be improved when GLO activities were increased by 60 or 100%, whereas reduced growth was noticed when the activity was further increased by 150 or 210%. As compared with WT plants, all the overexpression plants exhibited significantly improved Pn under conditions of high light and high temperature, but not under normal conditions. In addition, the overexpression plants were more resistant to the MV-induced photooxidative stress. It was further demonstrated that the antioxidant enzymes, and the antioxidant metabolite glutathione was not significantly altered in the overexpression plants. In contrast, H_2_O_2_ and salicylic acid (SA) were correspondingly induced upon the GLO overexpression. Taken together, the results suggest that GLO may play an important role for plants to cope with high light and high temperature, and that H_2_O_2_ and SA may serve as signaling molecules to trigger stress defense responses but antioxidant reactions appear not to be involved in the defense.

## Introduction

Photorespiration (PR) is the second highest metabolite flux only next to photosynthesis (Pn) in C3 plants, with flux rates amounting to 25–30% of Pn (Sharkey, 1988; Peterhansel and Maurino, 2011). The rate can be even higher under stress conditions such as high temperature, high light and CO_2_ or water deficit (Foyer et al., 2009; Peterhansel and Maurino, 2011). PR is also considered as a major source for hydrogen peroxide (H_2_O_2_) in plants, likely accounting for more than 70% of total cellular H_2_O_2_ in photosynthetic leaves of C3 plants (Noctor et al., 2002; Foyer et al., 2009; Peterhansel and Maurino, 2011). Cellular H_2_O_2_ is an important reactive oxygen species (ROS), which function as a signaling molecule to regulate various physiological and defense processes. While different sources for H_2_O_2_ have been reported in plants, the peroxisomal H_2_O_2_ has recently received increasing attention and shown to play important roles in the programmed cell death (PCD) and biotic defense responses (Chaouch and Noctor, 2010; Sewelam et al., 2014). The peroxisomal H_2_O_2_ is mainly contributed by the glycolate oxidation reaction catalyzed by glycolate oxidase (GLO) (Noctor et al., 2002; Foyer et al., 2009). As a result of this, physiological functions of GLO are often considered to link with H_2_O_2_ signaling (Chamnongpol et al., 1998; Peterhansel and Maurino, 2011). GLO could be induced in response to various environmental stresses, as noticed in Vigna, pea and tobacco (Mukherjee and Choudhuri, 1983; Mulligan et al., 1983; Mittler and Zilinskas, 1994; Rizhsky et al., 2002). GLO was also implicated in plant resistance to pathogens (Mukherjee and Choudhuri, 1983; Taler et al., 2004; Rojas et al., 2012; Gilbert and Wolpert, 2013). Taler et al. (2004) identified “enzymatic resistance” genes in melon and suggested that the enhanced expression of the peroxisomal serine/glyoxylate aminotransferase (SGAT) correlated with higher GLO activity which was proposed to play a role in the resistance to *Psilocybe cubensis* by greater production of H_2_O_2_ (Taler et al., 2004). It is more recently demonstrated that GLO is an alternative source for the production of H_2_O_2_ during both gene-for-gene and non-host resistance in *Nicotiana benthamiana* and *Arabidopsis* (Rojas et al., 2012; Gilbert and Wolpert, 2013).

In addition, GLO has been frequently implicated to markedly affect Pn, mainly through studies using mutants or RNAi transgenic plants (Yamaguchi and Nishimura, 2000; Xu et al., 2009; Zelitch et al., 2009; Lu et al., 2014). Interestingly, all these studies consistently found that GLO-deficient C3 plants, or even C4 maize, displayed typical PR phenotypes, i.e., the plants are lethal or stunted in air while normal under high CO_2_. This phenotype is similar to what was observed in mutants with defects of the other photorespiratory enzymes (Somerville, 2001; Boldt et al., 2005; Timm and Bauwe, 2013). The PR phenotype in the C4 maize *glo* mutant may implicate that either the photorespiratory pathway is equally important in C4 plants as in C3 plants (Zelitch et al., 2009), or that GLO plays a second essential, yet unidentified, role in plants, as once proposed by Somerville and Ogren (1982). More intriguingly, our previous work has shown that suppression of GLO led to inhibited Pn, which was positively and linearly correlated with the decreased GLO activities (Xu et al., 2009). A few studies have reported that increased levels of photorespiratory enzymes in plants improved Pn or even growth parameters (Timm et al., 2012, 2015, 2016). So it is of curiosity to further know if Pn can be improved when GLO is up-regulated? In this study, various GLO overexpression rice lines, with gradient increases in activity, were generated in order to address the above question. Further functional analyses on these plants indicate that GLO may play an important role for plants to cope with high light and high temperature, and that H_2_O_2_ and salicylic acid (SA) may serve as signaling molecules to trigger stress defense responses but antioxidant reactions appear not to be involved in the defense.

## Materials and Methods

### Growth Conditions and Treatments

Pre-germinated rice seeds and transgenic plants were normally grown in Kimura B complete nutrient solution (Yoshida et al., 1976) under natural conditions [average temperature of 30–35/23–26°C (day/night), photosynthetically active radiation 600–1500 μmol m^-2^ s^-1^ and photoperiod of 12 h day/12 h night]. The solution was adjusted to pH of 4.8–5.0 and was renewed once in a week. Various treatments are specified in the corresponding figure legends.

### Construction of the *GLO*-Overexpression Transgenic Rice Lines

Rice (*Oryza sativa* L. cv. Zhonghua 11) was used for constructing transgenic lines in this study. The complete cDNA sequences of *OsGLO1* (Os03g0786100) or *OsGLO4* (Os07g0152900) were amplified by RT-PCR, then the sequence was inserted into an overexpression vector named pYLox.5. PCR with specific primers and cutting with restriction enzymes proved that the target fragment had been correctly ligated. DNA sequencing finally confirmed the correct orientation and 100% cDNA identity to that reported in the GeneBank. The constructed vectors were then transformed into rice callus via *Agrobacterium*-mediated infection (strain EHA105). T_0_ lines were analyzed by Southern blot, and T1 seeds with a single T-DNA insertion were grown to produce T2 seeds. Homozygous lines were finally obtained with hygromycin-resistance screen.

### Transcript Analysis, Enzyme Activity and Metabolite Assays

#### Semi-quantitative and Real-Time PCR

Total RNA was isolated using TRIZOL reagent. The isolated total RNA was then further treated with DNase I and used as a template for first-strand cDNA synthesis using ReverTra Ace (Toyobo, Osaka, Japan) with random hexamers according to the manufacturer’s instructions. For semi-quantitative RT-PCR analysis, the optimal number of PCR cycles was first tested gene by gene. The PCR products were separated on 1% (w/v) agarose gels and visualized by Goldview staining. For real-time quantitative RT-PCR, the PCR reaction consisted of 10 μL of 2 × SYBR Green PCR Master Mix (Toyobo), 200 nM primers, and 2 μL of 1:40-diluted template cDNA in a total volume of 20 μL. No template controls were set for each primer pair. The analysis was conducted by a DNA Engine Option 2 Real-Time PCR Detection system and Opticon Monitor software (Bio-Rad, USA).

#### Enzyme Activity Assays

Glycolate oxidase activity was assayed according to Hall et al. (1985) with some modifications (Xu et al., 2009). Superoxide dismutase (SOD) activity was assayed by monitoring the inhibition of the photochemical reduction of nitroblue tetrazolium (NBT) according to the method of Beauchamp and Fridovich (1971), Catalase (CAT) activity was determined by following the consumption of H_2_O_2_ (extinction coefficient 43.6 M^-1^ cm^-1^) at 240 nm for 1 min (Aebi, 1984). The crude extract for guaiacol peroxidase (POD) measurements was isolated according to Polle et al. (1994). Ascorbate peroxidase (APX) activity was determined in the soluble fraction and in the chloroplast membrane fraction in 2 mL reaction mixture containing 50 mM potassium phosphate (pH 7.0), 0.5 mM ascorbate (extinction coefficient 2.8 mM^-1^ cm^-1^), 0.1 mM H_2_O_2_, and leaf extract causing a linear decrease in absorbance at 290 nm for 1 min (Nakano and Asada, 1981). Protein concentration was determined according to Bradford (1976).

#### MV Treatment

The youngest fully expanded leaves were detached and treated with 6 μM Methyl viologen (MV, *N*, *N*′-dimethyl-4, 4′-bipyridinium dichloride) at 30°C under continuous illumination (100 μmol m^-2^ s^-1^) for 0, 3, 6, 9, 12 h to induce photooxidative stress (Kim and Lee, 2002).

#### Metabolite Assays

Glutathione (GSH) and glutathione disulfide (GSSG) were determined according to Rahman et al. (2006). SA was measured according to Meuwly and Métraux (1993). SA was quantified fluorimetrically (G1321B scanning fluorescence detector, Agilent, USA), with excitation at 305 nm and emission at 407 nm. Hydrogen peroxide (H_2_O_2_) production was detected by staining with a freshly prepared 3, 3′-diaminobenzidine (DAB) solution (1 mg/ml, pH 3.8) for 2 h in light at 30°C. The experiment was terminated by boiling the leaves in ethanol for 30 min (Thordal Christensen et al., 1997).

### Gas Exchange Measurements and Chlorophyll Fluorescence analysis

Gas exchange characteristics including net photosynthetic rate (Pn), stomatal conductance (Gs) and internal CO_2_ concentration (Ci) were analyzed *in situ* using a portable Pn system (LI-6400, LI-COR). The plants were grown in normal natural condition or in an environment-controlled growth chamber, and the youngest fully expanded leaves were used to determine the photosynthetic parameters. Measurements were performed in the morning (10:00–12:00), unless specified elsewhere. The other conditions were set as follows: leaf temperature 30°C, humidity 60%, CO_2_ concentration 400 μmol mol^-1^, photosynthetic photon flux density (PFD) 1000 μmol m^-2^ s^-1^. For determining the curves of Pn versus PFD, light intensity was controlled by a LI-COR LED irradiation source.

The chlorophyll fluorescence was measured with a PAM 2100 portable chlorophyll fluorometer. Leaves were dark adapted for at least 20 min prior to the measurement. Two measurements were taken from each seedling to determine Fo and Fm, and the maximal photochemical efficency of PSII (Fv/Fm) was calculated according to Krause and Weis (1991).

### Statistical Analysis

The data were subjected to statistical analysis using Duncan’s multiple range test at the 5% (*P* < 0.05) confidence levels. Data Processing System (DPS) software (Tang and Zhang, 2013) were used for data statistics analysis.

## Results

### Generation of GLO Overexpression Rice Lines

**FIGURE 1:**
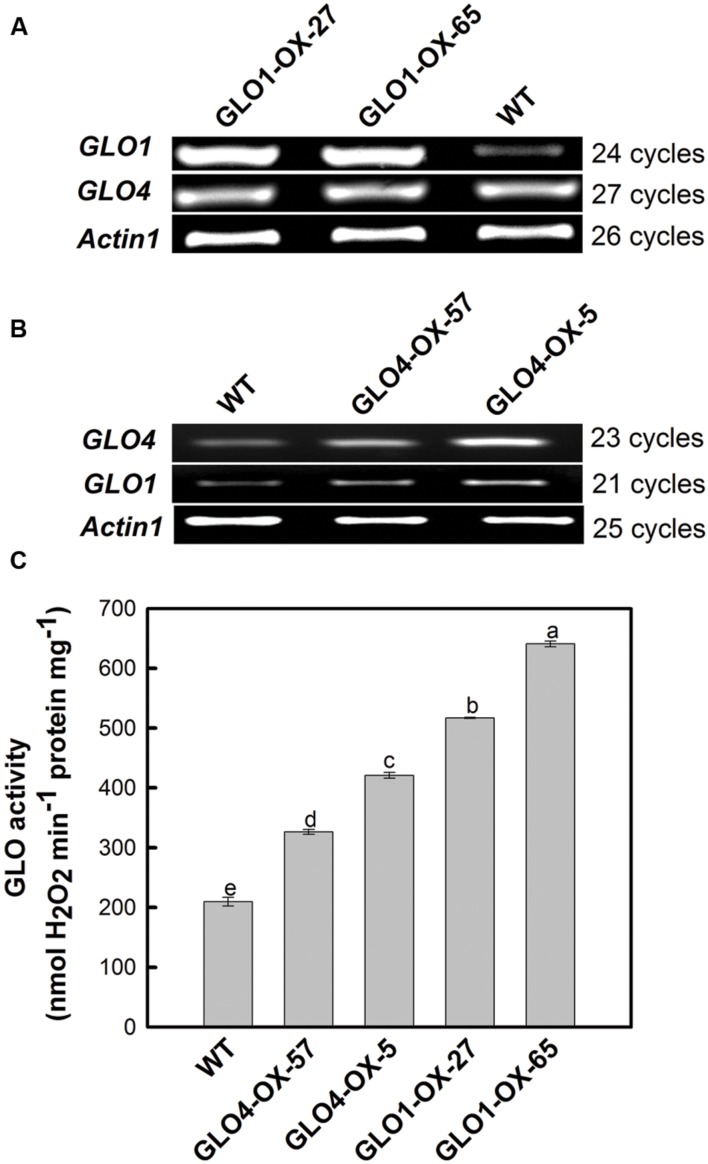
**Expressional verification of the glycolate oxidase (GLO) overexpression lines.** The plants were grown in Kimura B nutrient solution under normal natural conditions [temperature of 30–35/23–26°C (day/night), photosynthetically active radiation of 600–1500 μmol m^-2^ s^-1^ and photoperiod of 12 h day/12 h night]. The fully expanded leaf was detached at four-leaf stage for assay of transcripts **(A,B)** and activity **(C)**. The *OsActin* gene was used as an internal control. The data are means ± SD of three biological replicates, and representative of three independent experiments. Different letters on the top of columns indicate significant difference at *p* < 0.05 according to Duncan’s multiple range test.

Differential GLO overexpression rice lines were generated by upregulating either *GLO1* or *GLO4*. Four independent homozygous lines (two each for either *GLO1* or *GLO4*) were selected for this study. As shown in **Figure 1**, when GLO was upregulated at the mRNA level (**Figures 1A,B**), its catalytic activity was differentially increased, ranging from +60% to +210% (**Figure 1C**). Since we have previously demonstrated that GLO1 and GLO4 were responsible for controlling GLO catalytic activities and that specific silencing of either GLO1 or GLO4 exhibited same phenotypes, indicating both play same physiological roles in rice (Zhang et al., 2012). Thus, for further functional analyses, we shall be able to use these four independent transgenic rice lines with gradient activity increases by overexpressing either GLO1 or GLO4.

### Phenotypes of GLO Overexpression Lines

**FIGURE 2:**
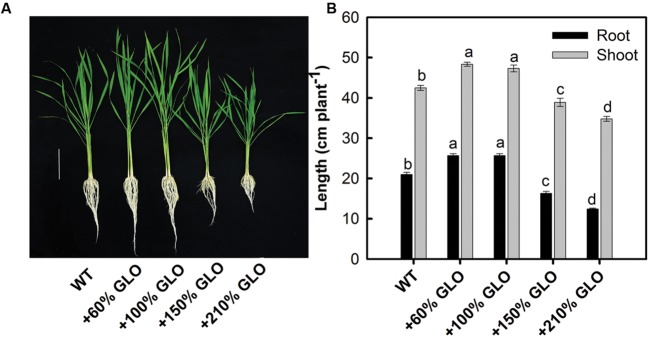
**Growth phenotypes of the GLO overexpression lines.** The plants were grown as described in **Figure 1.** **(A)** The picture was taken, and **(B)** growth of shoot and root was measured when the plants were grown to six-leaf stage. Bar in **(A)** = 10 cm. The other legends are the same as those in **Figure 1**.

Under normal natural conditions, the lines with 60 and 100% increases in GLO activities had significantly higher growth than WT (**Figure 2**). But, as the activity was further increased by 150 or 210%, the growth was inhibited (**Figure 2**).

### Photosynthesis of GLO Overexpression Lines under Normal and Stressful Conditions

**FIGURE 3:**
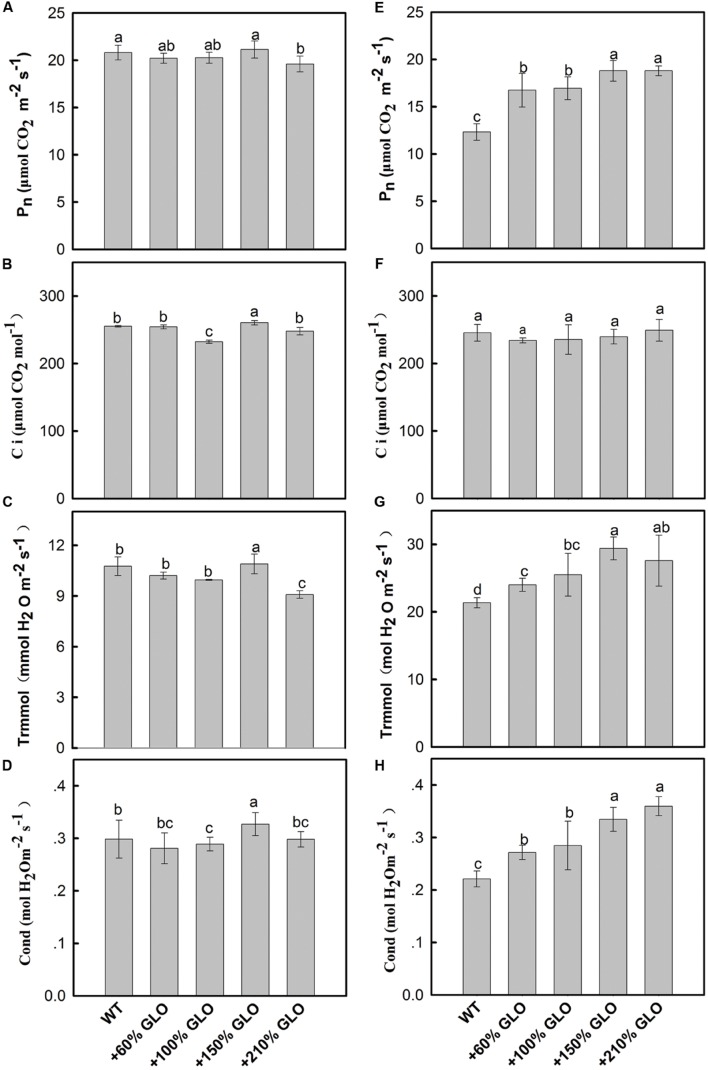
**Photosynthetic parameters of the GLO overexpression lines under normal natural conditions or high light and high temperature conditions.** The plants were grown under normal natural conditions as described in **Figure 1.** Until 5-leaf stage, net photosynthesis rates **(A)**, internal CO_2_ concentration **(B)**, transpiration rates **(C)**, and stomatal conductance **(D)** were measured using the youngest fully expanded leaves at between 10:00 and 12:00 of the day. The plants were transferred to a growth chamber with temperature of 40°C (day)/30°C (night) and light of 900 μmol m^-2^ s^-1^ and 65% humidity. At 3 days after the treatment, the photosynthetic parameters were determined in **(E–H)**. The data are means ± SD of four measurements on different plants and representative of three independent experiments. The other legends are the same as those in **Figure 1**.

**FIGURE 4:**
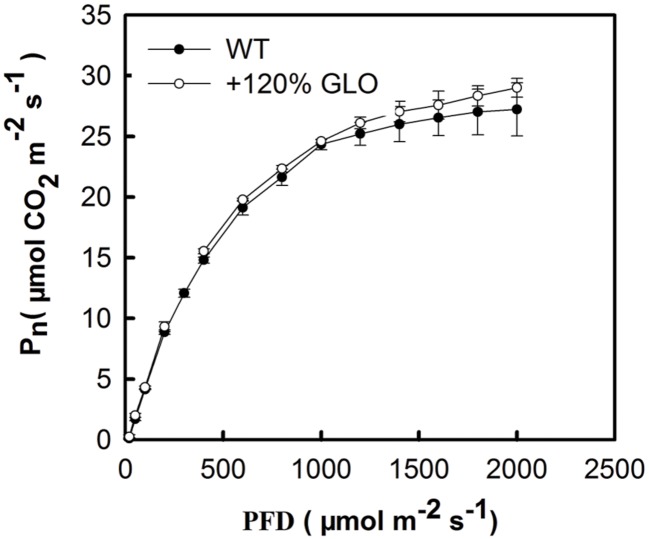
**Response of photosynthesis (Pn) to light intensity.** The plants were grown under natural conditions as described in **Figure 1.** Until six-leaf stage, the Pn was determined with different light intensity. The other legends are the same as those in **Figure 3**.

Under normal natural conditions, as has been previously reported, the photosynthetic rate (Pn) was heavily inhibited if GLO was suppressed in either high photorespiratory C3 or low photorespiratory C4 plants (Xu et al., 2009; Zelitch et al., 2009; Lu et al., 2014). More intriguingly, a positive and linear correlation was noticed between Pn and GLO activities when the enzyme was differentially down-regulated by an inducible antisence approach (Xu et al., 2009). Thus, we are curious to know whether Pn can be improved when GLO is upregulated. Here, we generated differential GLO overexpression lines to study their photosynthetic performance. Unexpectedly, all the GLO overexpression lines showed no preference in photosynthetic capacities under normal natural conditions as compared with WT (**Figures 3A–D**). However, measurements of the Pn response to light intensity pointed to a tendency that the overexpression plants may have photosynthetic preference under high light conditions because, as light intensity was increased to high levels (over 1200 μmol m^-2^ s^-1^), Pn in WT became leveled off (light saturation point) while it was still gradually increased in the overexpression plants (**Figure 4**).

Under high light and high temperature conditions. We further tested whether differences may occur under stressful conditions. The plants were first grown in a greenhouse under normal natural conditions, then transferred to a growth chamber with temperature of 40°C (day)/30°C (night) and light intensity of 900 μmol m^-2^ s^-1^. Three days after the treatment, photosynthetic parameters were determined. As shown (**Figures 3E–H**) Pn, transpiration rate and stomatal conductance were all significantly improved in the overexpression lines as compared with WT plants, except that the internal CO_2_ concentration stayed unaltered for all the plants.

**FIGURE 5:**
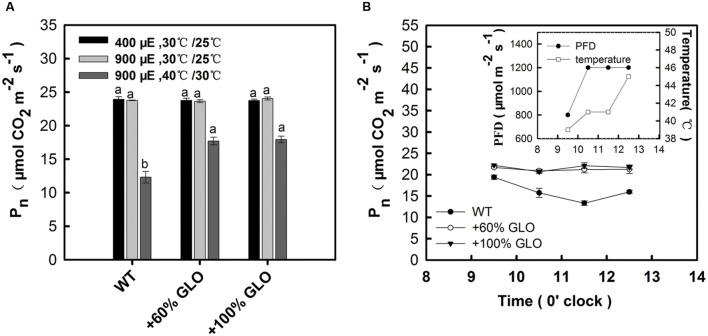
**A further test for Pn of the GLO overexpression lines under high light and high temperature conditions.** **(A)** The plants were first grown under light of 400 μmol m^-2^ s^-1^ and temperature of 30°C (day)/25°C (night) in a growth chamber to five-leaf stage, then treated with two conditions: (i) light of 900 μmol m^-2^ s^-1^ and temperature of 30°C (day)/25°C (night); (ii) 900 μmol m^-2^ s^-1^ and 40°C (day)/30°C (night). At 3 days after the treatment, Pn was determined. **(B)** The plants were first grown to five-leaf stage in a greenhouse during summer season. The temperature was artificially increased by attenuating the air circulation, where temperature and light can be quickly increased to high levels during the noon (see the inset). At 3 days after such condition, Pn was determined. “μE” represents “μmol m^-2^ s^-1^.” The data are means ± SD of four measurements on different plants and representative of three independent experiments. The other legends are the same as those in **Figure 1**.

A comparative study was further conducted to verify the above results. The plants were first grown under light of 400 μmol m^-2^ s^-1^ and temperature of 30°C (day)/25°C (night) in a growth chamber, and then treated with two conditions: (i) light of 900 μmol m^-2^ s^-1^ and temperature of 30°C (day)/25°C (night); (ii) 900 μmol m^-2^ s^-1^ and 40°C (day)/30°C (night). The results found that the overexpression plants had significantly higher Pn than WT plants only under the high temperature plus high light conditions, but not different under only this high light (900 μmol m^-2^ s^-1^) (**Figure 5A**). For further reinforcement, another experiment was carried out under a natural condition. The plants were grown in a greenhouse during summer season. The temperature was artificially increased by attenuating the air circulation, where temperature and light could be quickly increased to high levels during the noon (**Figure 5B** inset). At 3 days after such condition, Pn was determined. As the temperature and light intensity were increased during the day, Pn of WT plants was decreased while it remained stable for the overexpression plants (**Figure 5A**), further demonstrating photosynthetic preference for the overexpression plants under high light and high temperature conditions.

### Resistance of GLO Overexpression Lines to MV-Induced Oxidative Stress

**FIGURE 6:**
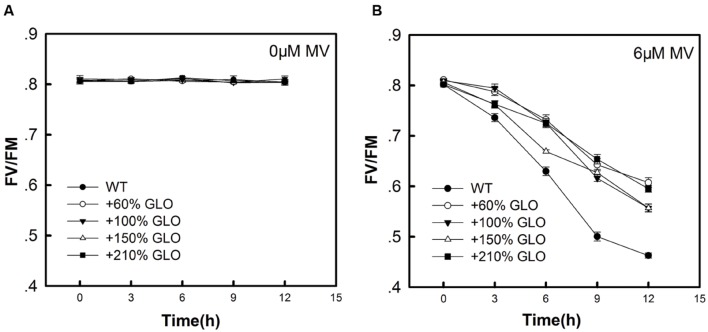
**Fv/Fm of the overexpression plants as treated by MV.** When the plants were grown to six-leaf stage, the youngest fully expanded leaves were detached and treated with 0 μM Methyl viologen (MV) **(A)** or 6 μM MV **(B)** under 100 μmol m^-2^ s^-1^ of light intensity. Fv/Fm was measured at different time points (0, 3, 6, 9, and 12 h). The data are means ± SD of four biological replicates and representative of three independent experiments.

MV is known to be able to induce oxidative stress in plants, particularly under photosynthetic conditions (Kim and Lee, 2002). In addition, MV is also reported to inhibit cyclic electron flow that is essential for photoprotection (Fan et al., 2007, 2008). As shown in **Figure 6**, when the detached rice leaves were treated with MV, all the GLO overexpression lines showed more resistance than WT plants to the MV-induced photooxidative stress.

### H_2_O_2_ and SA Accumulation in Response to GLO Overexpression

**FIGURE 7:**
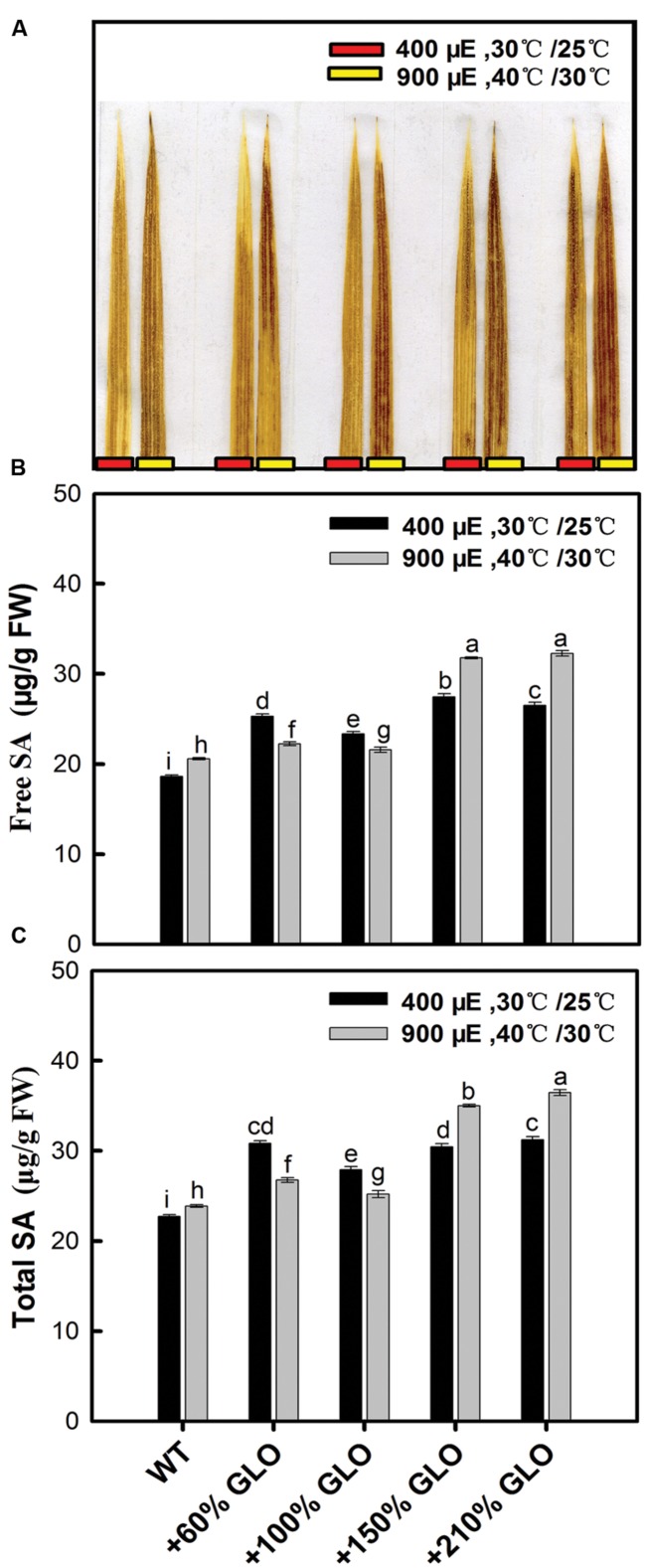
**H_2_O_2_ and salicylic acid (SA) accumulation in the GLO overexpression lines.** Five-leaf plants were grown in a growth chamber under a normal condition [30°C (day/12 h)/25°C (night/12 h), 400 μmol m^-2^ s^-1^ light intensity, and 65% humidity] for 3 days, then the temperature and light intensity were increased to 40°C (day/12 h)/30°C (night/12 h) and 900 μmol m^-2^ s^-1^, respectively, for 24 h. H_2_O_2_ and SA were measured before and after the treatments. **(A)** H_2_O_2_-3′-diaminobenzidine (DAB) staining and the result is representative of three independent experiments; **(B,C)** SA was determined by HPLC chromatography. The other legends are the same as those in **Figure 1**.

As described previously, GLO is always linked to the photorespiratory H_2_O_2_ production in plants. Here we estimated the H_2_O_2_ content in rice leaves by DAB staining. As shown in **Figure 7A**, H_2_O_2_ was increased in all the GLO overexpression lines under both normal and stressful conditions. It has been documented that H_2_O_2_ and SA may function together in a self-amplifying feedback loop, in which H_2_O_2_ induces SA accumulation and SA in turn enhances H_2_O_2_ accumulation (Chaouch and Noctor, 2010; Miura et al., 2013; Xia et al., 2015). So we further determined the response of SA to the GLO overexpression. The results showed that the contents of both free and total SA were increased in all the overexpression lines compared with WT, similar to the H_2_O_2_ accumulation.

### Responses of Antioxidant Reactions to GLO Overexpressions

**FIGURE 8:**
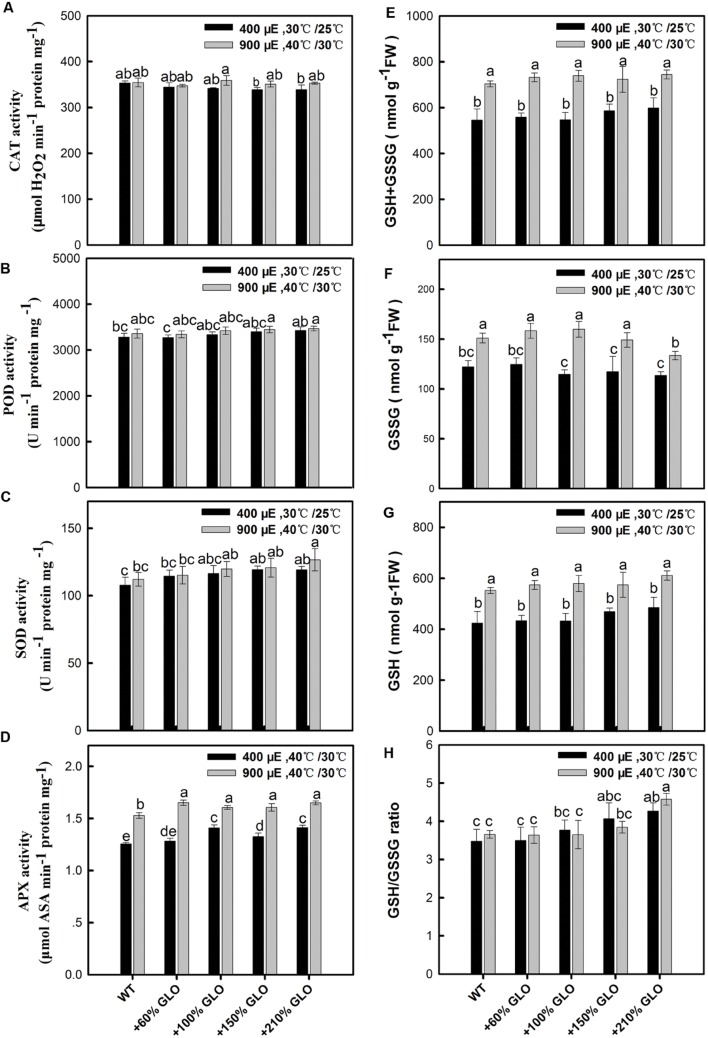
**Effects of GLO overexpressions on antioxidant enzymes and glutathione contents.** The legend is the same as that in Figure. Activity analysis of antioxidant enzymes **(A–D)**, including CAT, POD, SOD, and APX. **(E)** Concentration of total glutathione (GSH + GSSG), **(F)** GSSG, **(G)** GSH and the ratio of GSH/GSSG **(H)**. The other legends are the same as those in **Figure 1**.

As noticed above, H_2_O_2_ was increased in all the GLO overexpression lines under both normal and the stressful conditions, so it is interesting to know if the antioxidant defense reactions are activated by the increased H_2_O_2_. Unexpectedly, the antioxidant enzymes such as SOD, CAT, POD, and APX, were little altered in the overexpression lines compared with WT plants and the antioxidant metabolite glutathione was also not affected by overexpressing GLO in rice (**Figure 8**).

## Discussion

Previous results have demonstrated that photosynthetic inhibition occurred either in C3 or C4 plants if GLO was suppressed (Xu et al., 2009; Zelitch et al., 2009; Lu et al., 2014). More intriguingly, a positive and linear correlation was noticed between GLO activities and Pn in rice (Xu et al., 2009). Thus we are curious about what will occur and whether Pn can be improved when GLO is upregulated (Timm et al., 2016). In order to address this question, we further generated differential GLO everexpression rice lines and then conducted detailed functional analyses on these plants, including phenotypic, physiological and biochemical analyses. Phenotype observations showed that, under normal conditions, the lines with 60 or 100% increase in GLO activity showed improved growth whereas the lines with further increases (+150% or +210%) conferred reduced growth (**Figure 2**). But, under normal conditions, photosynthetic parameters were not improved in all these overexpression lines (**Figure 3**). It appears that the improved growth for the first two lines is not correlated with the Pn, but possibility still exists that Pn may have been transiently improved sometimes during the whole growth stage under normal conditions, which failed to be detected by our limited time-point measurements.

Measurements on the Pn response to light intensities pointed to a tendency that the GLO overexpression plants have photosynthetic preference under high light conditions (**Figure 4**). This led us to further test the photosynthetic performance under stressful conditions. Resultantly, under conditions of high light plus high temperature, photosynthetic capacities were significantly improved in the overexpression plants (**Figures 3** and **5**). Moreover, the overexpression plants were more resistant to the MV-induced photo-oxidative stress than WT plants (**Figure 6**). These results collectively suggest that GLO may play a critical role for Pn to cope with high light plus high temperature or the induced oxidative stress. Pn is known as the most sensitive physiological process to stresses, and any alterations in photosynthetic attributes under stresses are good indicators of the plant stress tolerance, and thus, in any species the ability to sustain leaf gas exchange under stress has direct relationship with the stress tolerance (Wahid et al., 2007). In other words, it may be extended that GLO may play important roles for rice plants to cope with high light and high temperature, or the induced oxidative stress. Such a role is of far-reaching practical significance as rising atmospheric CO_2_ is driving temperature increases (i.e., global warming) in many already stressful environments, such as strong light and drought, particularly for rice, as a staple food crop (Singh et al., 2014).

Photorespiration is generally stimulated as light intensity is increased (Brown and Morgan, 1980; Gerbaud and André, 1980; Vines et al., 1982; Haupt-Herting et al., 2001), which is even more dependent on light intensity when coupled with other stresses, such as high temperature, water stress or CO_2_ deficit (Kangasjärvi et al., 2012). High temperature can stimulate photorespiratory flux even if light intensity is constant, because (i) the solubility of CO_2_ in water decreases with temperature more than the solubility of O_2_, resulting in a lower CO_2_:O_2_ ratio at the active site of Rubisco (ribulose-1, 5-bisphosphate carboxylase/oxygenase); and (ii) the enzymatic properties of Rubisco shift with temperature, stimulating RuBP oxygenation to a greater degree than RuBP carboxylation (Foyer et al., 2009). In addition, high temperature and high light can result in stomatal closure, which reduces the C:O ratio around Rubisco, thereby promoting PR as an indirect result (Kangasjärvi et al., 2012). Thus, high light plus high temperature may be able to markedly stimulate photorespiratory metabolism, leading to the overproduction of glycolate. If such glycolate is not removed timely and accumulated within chloroplasts, it may be converted into glyoxylate by a possibly existing photosystem I-dependent oxidation system (Murai and Katoh, 1975; Goyal and Tolbert, 1996; Goyal, 2002). The accumulated glyoxylate in chloroplasts has been known to inhibit Pn (Chastain and Ogren, 1989; Campbell and Ogren, 1990; Lu et al., 2014). Under such circumstances, therefore, a higher level of peroxisomal GLO is able to facilitate a timely scavenging of the overproduced glycolate so as to avoid its toxicity to chloroplasts.

In contrast with the above notion, Nölke et al. (2014) recently reported that promoting glycolate oxidation within chloroplasts even improved Pn and yield in potato (Nölke et al., 2014). In addition, the above notion may not explain the result that the overexpression lines show more resistance to the MV-induced photooxidative stress (**Figure 6**). It has been demonstrated that GLO plays important roles in both biotic and abiotic responses or resistance (Mukherjee and Choudhuri, 1983; Bohman et al., 2002; Taler et al., 2004; Rojas et al., 2012). Considering mechanisms, the researchers always link it to the GLO-catalyzed H_2_O_2_ production, as is known to play a signaling role in various physiological processes (Foyer et al., 2009). It is extensively documented that H_2_O_2_ originates mainly in apoplasts associated with the plasmalemma, but evidences are accumulating to show that other intracellular sources of H_2_O_2_, notably chloroplasts, peroxisomes and mitochondria, could be also involved. Peroxisomes and chloroplasts may accumulate 30–100 times higher H_2_O_2_ as compared to mitochondria (Hossain et al., 2015). The peroxisomal H_2_O_2_ is well known to be ultimately contributed by the GLO-catalyzed glycolate oxidation (Noctor et al., 2002; Foyer and Noctor, 2003; Kangasjärvi et al., 2012). Accumulation of the peroxisomal H_2_O_2_ stimulated the isochorismate-dependent SA synthesis and then triggered SA-related pathogenesis responses and defense gene expressions in plants (Chamnongpol et al., 1998; Chaouch et al., 2010; Kangasjärvi et al., 2012). The peroxisomal H_2_O_2_ can also induce oxidative stress that would activate programme cell death (PCD) under long day and high light if not controlled by CAT activity (Chaouch et al., 2010; Mhamdi et al., 2010; Suzuki et al., 2011). Sewelam et al. (2014) most recently revealed that the peroxisomal H_2_O_2_ induced transcripts for stress tolerance, and Rojas et al. (2012) presented more strong evidence indicating that the GLO-catalyzed H_2_O_2_ production contributed to both gene-for-gene and non-host resistance in *Nicotiana benthamiana* and *Arabidopsis*.

In this study, we observed that both H_2_O_2_ and SA were correspondingly induced but the antioxidant reactions were not responsive upon the GLO overexpression (**Figures 7** and **8**), although the result that the overexpression lines are more resistant to the MV-induced photooxidative stress (**Figure 6**) points toward possibilities that the antioxidant systems have been activated in these plants. While many publications have demonstrated that exogenous or stress-induced H_2_O_2_ is able to activate the antioxidant defense system, including both non-enzymaitc and enzymatic, the correlation between the endogenous H_2_O_2_ and antioxidant systems is not well established so far (Neill et al., 2002; Winfield et al., 2010; Del, 2015). During the past years, by using CAT-deficient mutants and/or GLO-upregulated transgenic plants as an endogenous H_2_O_2_ burst producer, it was only observed that some antioxidant enzyme genes, such as APX and GPX, were induced at both transcript and protein levels as the endogenous H_2_O_2_ is enhanced, but few data at the activity level confirmed these responses (Neill et al., 2002; Mhamdi et al., 2010; Sewelam et al., 2014; Del, 2015; Xia et al., 2015). It seems that exogenous or stress-induced H_2_O_2_ could be different from endogenous H_2_O_2_ in triggering metabolic or physiological responses, likely the former being mostly a stressor while the latter acting mostly as a signal. In contrast, the results that H_2_O_2_ and SA were correspondingly induced in the overexpression plants (**Figure 7**) are in well agreement with previous results (Chaouch et al., 2010; Mhamdi et al., 2010). These two substances have been well known as key signaling molecules to be able to trigger various defense responses (Herrera-Vásquez et al., 2015; Xia et al., 2015), both of which may function together in a self-amplifying feedback loop, in which H_2_O_2_ induces SA accumulation and SA in turn enhances H_2_O_2_ accumulation (Khokon et al., 2011; Miura et al., 2013; Xia et al., 2015). Therefore, it can be inferred that both H_2_O_2_ and SA are involved in triggering some stress defense responses, but not including antioxidant reactions, for the GLO overexpression plants to cope with high light and high temperature.

## Author Contributions

X-XP conceived the idea and designed the experiments. L-LC, Y-sL, and YL performed the experiments. X-XP wrote the manuscript. CY and L-LC edited the manuscript. All the authors approved the final manuscript.

## Conflict of Interest Statement

The authors declare that the research was conducted in the absence of any commercial or financial relationships that could be construed as a potential conflict of interest.
